# Density Functional Theory-Assisted Synthesis of Self-Curing Epoxy–Acrylic Resin

**DOI:** 10.3389/fchem.2020.595954

**Published:** 2021-01-20

**Authors:** Bowen Yang, Qiufeng An

**Affiliations:** Shaanxi Key Laboratory of Chemical Additives for Industry, Shaanxi University of Science and Technology, Xi'an, China

**Keywords:** density functional theory, the free radical reaction index, self-curing, quantum chemistry reaction index, free radical reaction index

## Abstract

A density functional theory-assisted synthesis of self-curing epoxy–acrylic resin (EMPA) is described. The calculated quantum chemistry reaction index of the reacting monomer in the basic state, i.e., the radical reaction index (*F*_*k*_^0^), is used as a guide to optimize the synthesis conditions. The reliability of the *F*_*k*_^0^-assisted synthesis method is confirmed after evaluating the physical appearance, mechanical properties after curing, and thermal stability of the obtained EMPA. The special functional groups of the resin are characterized by Fourier transform infrared spectroscopy to prove the rationality of the reaction mechanism. The cross-sectional morphology characteristics of the cured resin are observed by field-emission scanning electron microscopy. The results show that the closer the molar ratio of monomers in the reaction to the ratio of *F*_*k*_^0^ of the reacting monomers, the better the polymerization performance.

## Introduction

Common resins in daily life can be roughly divided into acrylic resins and epoxy resins (Wei et al., 2019). Acrylic resins have the advantages of strong adhesion and good film formation; however, they exhibit poor corrosion resistance (Yufeng et al., 2019). In contrast, epoxy resins have good corrosion resistance, excellent dimensional stability, and high insulation (Celikbag et al., 2017), which overcome the disadvantages of acrylic resins. In practical applications, the combination of the two resins by blending or piling up layer by layer can be expected to incorporate the advantages of the individual components. Since both types of resins require different curing agents, the selection of the curing agent may be challenging (Liu et al., 2019). Therefore, synthesizing a composite resin with the characteristics of epoxy and acrylic resins while achieving single-component curing would greatly reduce the cost of resin production and increase the profit in actual production (Tang et al., 2017).

In the process of synthesizing acrylic resin by free-radical polymerization, functional monomers with olefin groups and epoxy groups are used as reactants. After introduction of the epoxy group, a small anhydride molecule as the latent epoxy resin curing agent is incorporated into the acrylic resin following the same procedure used for the synthesis of a self-curable acrylic resin with epoxy groups. The ratio between the reactive monomers in the free-radical polymerization is of great significance for controlling the structure and properties of the polymer; different ratios lead to different structures, with concomitantly diverse polymer properties (Han et al., 2020). Therefore, a fast optimization of the ratio of reactants can improve the reaction efficiency and alleviate the hidden costs of continuous experimentation in actual production.

In recent years, with the development of density functional theory (DFT), the application of computational chemistry in chemical synthesis has become increasingly extensive (Chen et al., 2016; Guo et al., 2020). However, DFT is mostly used for the synthesis of small molecules; the determination of the chemical reactivity index of each atom in the target molecule allows to quantitatively design the reaction between molecules, thereby improving the reaction efficiency (Kaviani et al., 2016; Xiyuan et al., 2020). However, the application of DFT in the synthesis of polymers is rare (Shen et al., 2017; Yang et al., 2019). The radical reactivity index (*F*_*k*_^0^) of each atom in a molecule in the ground state can be quantitatively obtained using the DFT-developed Fukui function. This could be extrapolated to the synthesis of polymers; the *F*_*k*_^0^ of monomers participating in a free-radical polymerization could be obtained quantitatively by DFT calculation, which could enable optimization of the ratio of the reacting monomers for an enhanced polymerization efficiency.

This study describes a polymer synthesis guided by DFT calculation. The *F*_*k*_^0^ of each reaction monomer obtained by computer simulation is used to optimize the proportion of monomers involved in the reaction and to elucidate the mechanism of the free-radical polymerization reaction for the synthesis of self-curing epoxy–acrylic resin (EMPA). Finally, the analysis of the physical appearance, mechanical properties after curing, and thermal stability of the as-obtained resin confirms the reliability of the *F*_*k*_^0^-assisted synthesis of EMPA.

## Materials and Methods

### Materials

Butyl acrylate (BuAA), styrene (ST), maleic anhydride (MA), propenyl glycidyl ether (AGE), thioglycolic acid (TGA), and 2,2′-azobis-isobutyronitrile (AIBN) were purchased from Maclean (China) Chemical Reagent Co., Ltd. (Guangzhou, China). Butyl acetate and toluene were obtained from Tianjin Tianli Reagent Co., Ltd. (Tianjin, China). Diglycidyl ether of bisphenol A (DGEBA) (E51, Shanghai Resin Production, epoxy value 0.51 mol/100 g) was used as the epoxy resin used in this study.

### Synthesis of Self-Curing Epoxy–Acrylic Resin

**Figure 1:**
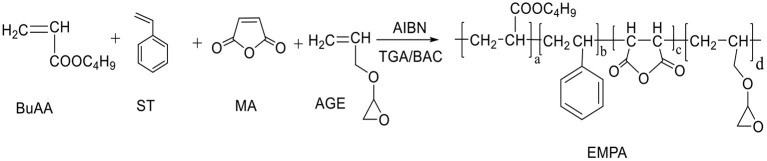
Polymerization process of epoxy–acrylic resin (EMPA).

**Figure 2:**
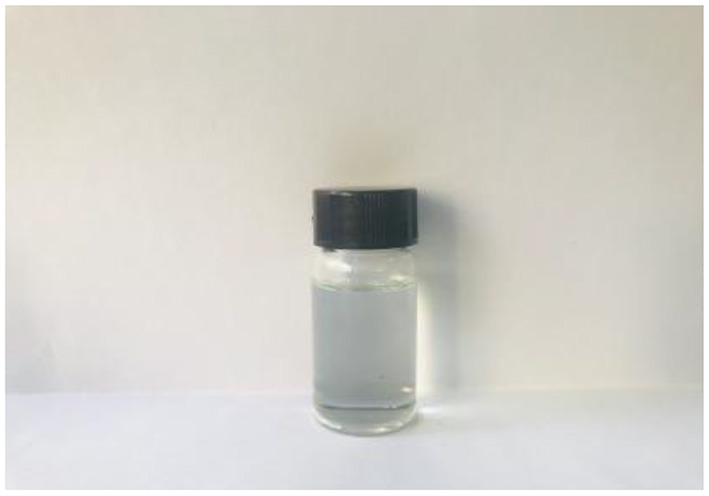
Appearance of epoxy–acrylic resin (EMPA).

BuAA, ST, propenyl glycidyl ether (AGE), and MA were dissolved in a toluene/butyl acetate solution according to the ratio described in Table 1 to prepare a 28% solution. The free-radical polymerization proceeded as shown in Figure 1. Briefly, 1% of the total monomer mass of AIBN was added and allowed to react for 30 min; then, 1% of the total monomer mass of TGA was added. After heating and stirring for 2 h at 80°C, 1 ml of H_2_O was added, followed by heating and stirring for further 30 min. Removal of the solvent at −0.1 MPa and 50°C afforded EMPA as a light yellow liquid (Figure 2).

**Table 1:** Designed molar ratio of the four monomers.

	**Butyl acrylate**	**Styrene**	**Propenyl glycidyl ether**	**Maleic anhydride**
1	0.1 mol	0.1 mol	0.05 mol	0.03 mol
2	0.1 mol	0.1 mol	0.05 mol	0.04 mol
3	0.1 mol	0.1 mol	0.05 mol	0.05 mol

### Characterization

Special functional groups were detected using a Fourier transform infrared (FTIR) spectrometer, and scanning electron microscopy (SEM) images were obtained using a field-emission SEM (SU8100). Thermogravimetry/derivative thermogravimetry (TG/DTG) analysis was performed using a thermal gravimetric analyzer Q500 with a nitrogen flow rate of 20 ml/min and a temperature range from 40 to 600°C at a heating rate of 10°C/min.

### Density Functional Theory Calculation of the Radical Reaction Index of Each Reactive Monomer

Gaussian09 software with DFT/b3lyp/6-311G++ (2d, 2p)/natural bond orbital (NBO) was used as basis set to structurally optimize the studied molecules. On this basis, the single-point energy of the neutral molecules and the energy of molecules that lose and gain an electron were calculated to obtain the relevant NBO charge data and the Wiberg bond order from the Wiberg bond index matrix in the NBO basis of the Gaussian09 output file.

The condensed Fukui functions $f_{k}^{\alpha }$ and the condensed dual descriptor of atoms in each molecule ($\Delta f_{k}^{\pm }$) can be obtained according to conceptual DFT (CDFT), where α is +, –, or 0; and k is the atomic serial number.

Condensed electrophilic Fukui function: $f_{k}^{+}=q_{k}^{N}-q_{k}^{N+1}$

Condensed nucleophilic Fukui function: $f_{k}^{+}=q_{k}^{N-1}-q_{k}^{N}$

Condensed Fukui function of free radical: $f_{k}^{0}=-\frac{1}{2}\left(f_{k}^{+}+f_{k}^{-}\right)=\frac{1}{2}\left(q_{k}^{N+1}-q_{k}^{N-1}\right)$

Condensed dual descriptor: $\Delta f_{k}^{\pm }=f_{k}^{+}-f_{k}^{-}$

where $q_{k}^{N}$, $q_{k}^{N+1}$, and $q_{k}^{N-1}$ are the atomic charge k of the neutral molecule, the atomic charge k of the molecule gaining one electron, and the atomic charge k of the molecule losing one electron, respectively, which were obtained by Gaussian09 calculation.

It should be noted that $f_{k}^{\alpha }$ and $\Delta f_{k}^{\pm }$ can only be used for comparison of the reactive strength of individual atoms within a molecule. This is because $\sum f_{k}^{\alpha }=1$, whereas $\Delta f_{k}^{\pm }$ is derived from the Fukui function with $\sum \Delta f_{k}^{\pm }=0$. Different molecules cannot be compared with the values of $f_{k}^{0}$ and $\Delta f_{k}^{\pm }$ because the total number of valence electrons involved in each reaction is different.

In addition, only valence electrons participate in the chemical reaction, irrespective of the type of reaction, i.e., electrophilic, nucleophilic, or free-radical reaction. For the main group elements, the reaction involves the outermost electron. According to the principle of quantum state superposition, the total number of electrons participating in the reaction in each molecule (NVe) can be multiplied by the corresponding index to provide comparison between molecules. NVe represents the sum of the number of valence electrons of each atom in a molecule. Therefore, the radical reaction index Fk0 and the dual descriptor reaction index $\Delta f_{k}^{\pm }$ were used for the description.

$$\begin{array}{l} \Delta {\text{F}}_{k}^{\pm }=N_{Ve}\Delta f_{k}^{\pm }\\ F_{k}^{0}=N_{Ve}\times f_{k}^{0} \end{array}$$

$\Delta {\text{F}}_{k}^{\pm }>$ 0 indicates that the k atom is sensitive to the attack of nucleophiles; that is, the atom is electrophilic. $\Delta {\text{F}}_{k}^{\pm }<0$ indicates that the k atom is sensitive to the attack of electrophiles; that is, the atom is nucleophilic.

Therefore, comparing the four free-radical reaction indices of the above four reaction monomers, the reactivity of the four materials in the current free-radical polymerization reaction follows the order BuAA > ST > AGE > MA.

According to the numerical ratio of the radical reaction indices (12.06972:10.36100:5.14510:3.96108), it was estimated that the highest polymerization efficiency was achieved for a molar ratio BuAA:ST:AGE:MA of 1:1:0.5:0.4. To verify the rationality of the free-radical reaction index influencing the monomer participation in the free-radical polymerization, three monomer ratios were designed as summarized in Table 1. By testing the mechanical properties of the synthetic resins in the three schemes (see Table 2), the scientificity of the calculated ratio is verified.

**Table 2:** Mechanical properties of three epoxy–acrylic resin (EMPA) resins.

	**1**	**2**	**3**
Elongation at break (%)	2.8	2.9	2.6
Tensile strength (MPa)	64	64.5	62.5
Bending strength (MPa)	110.8	110	109.5
Hardness (level)	3H	4H	4H

## Results and Discussion

### Self-Curing Mechanism of Epoxy-Acrylic Resin

**Figure 3:**
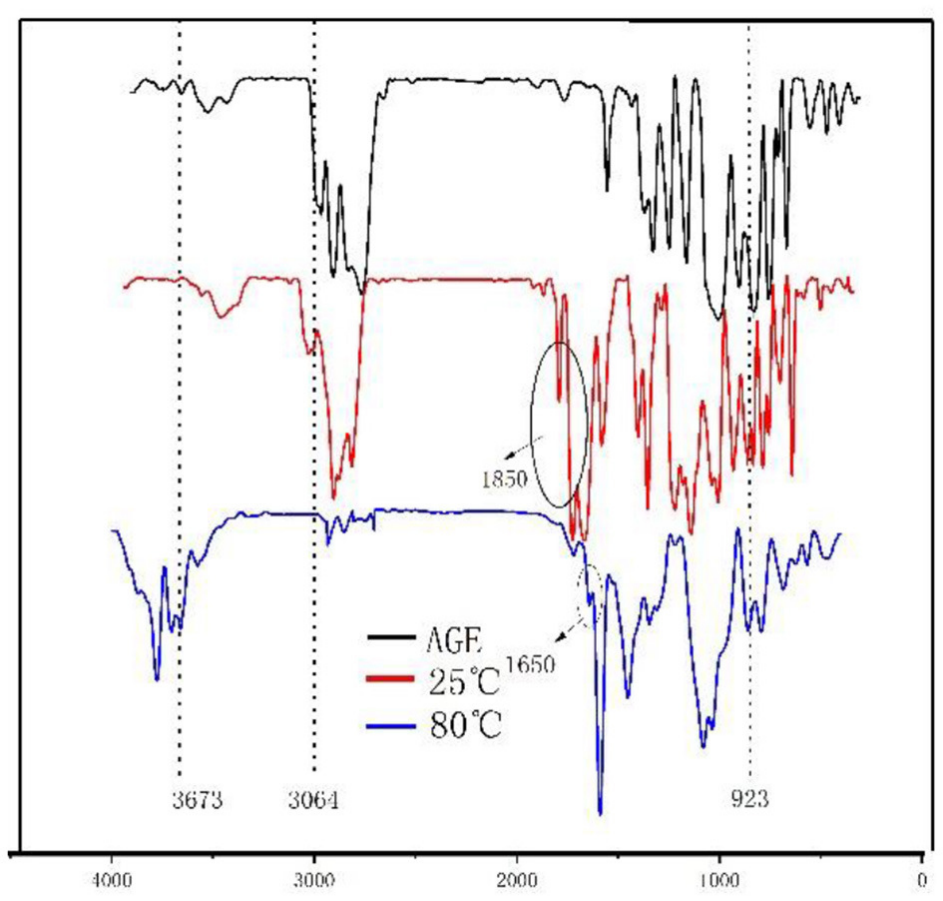
Infrared analysis diagram of each reaction stage of monomer and epoxy–acrylic resin (EMPA).

As shown in Figure 3, the monomers participating in the reaction form a linear structure through radical polymerization. The epoxy groups and anhydride groups extend from the linear molecular chain like antennae. Under the action of H^+^ and high temperature, cross-linking is completed and self-curing is completed.

### Fourier Transform Infrared Analysis of Epoxy–Acrylic Resin

As shown in Figure 3, the FTIR spectra of EMPA exhibit a vibration absorption peak attributable to the epoxy group at 923 cm^−1^ at 25 and 80°C. The C-O vibration absorption peak of the cyclic acid anhydride group at 1,850 cm^−1^ is observed at 25°C; however, it disappears at 80°C. Instead, a C-O vibration absorption peak of the carboxylic acid group appears at 1,650 cm^−1^. The disappearance of the C–H vibration absorption peak at 3,064 cm^−1^ after the reaction confirms that the radical polymerization reaction reaches completion. A vibration absorption peak of the –OH of the open anhydride can be observed at 3,673 cm^−1^ at 80°C. Taken together, these observations show that the free-radical polymerization reaction was complete after for 4 h at 80°C, there were epoxy groups in the polymer, and the acid anhydride group was ring-opened to produce carboxylic acid.

### Free-Radical Reaction Index $F_{k}^{0}$ and the Dual Descriptor Reaction Index $\Delta F_{k}^{\pm }$ Quantitative Description Atom in the Monomer Participates in the Free-Radical Polymerization Reaction

**Figure 4:**
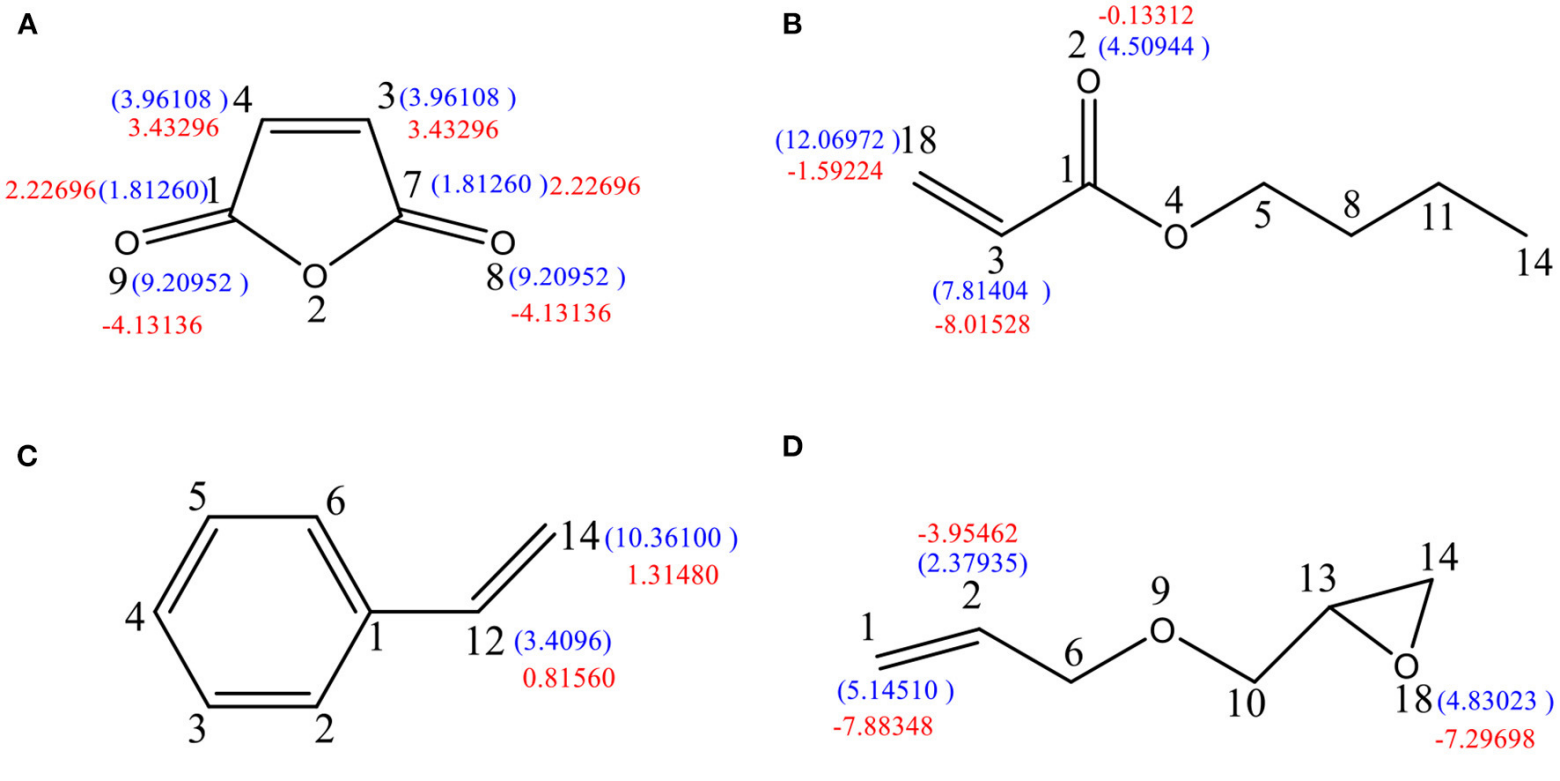
Free-radical reaction index and dual descriptor reaction index of active sites in the reactive monomer. **(A)** Maleic anhydride, **(B)** butyl acrylate, **(C)** styrene, and **(D)** propenyl glycidyl ether.

As shown in Figure 4A, the free-radical reaction indices of the C3, C4, O8, and O9 atoms were determined to be $F_{3C}^{0}$ = $F_{4C}^{0}$ = 3.96108 and $F_{8O}^{0}$ = $F_{9O}^{0}$ = 9.20952. Therefore, the four atoms are susceptible to participate in free-radical polymerization, with O8 and O9 being more prone to undergo free-radical reactions. However, two pairs of electrons of the oxygen atoms on the niobium base are involved in the sp^2^ hybridization of the carbon atoms, forming a stable carbon–oxygen double bond that prevents formation of a free radical with O8 and O9. This suggests that the free-radical reaction can only occur on C3 and C4. In addition, $\Delta F_{3C}^{\pm }=\Delta F_{4C}^{\pm }=$ 3.43296, $\Delta F_{8O}^{\pm }=\Delta F_{9O}^{\pm }=\;$-4.13136, and $\Delta F_{2O}^{\pm }=$ −2.37312. The values $\Delta F_{3C}^{\pm }=\Delta F_{4C}^{\pm }>0$ indicate the sensitivity of the C3 and C4 atoms to nucleophilic reagents, which render them prone to undergo electrophilic addition reactions. In contrast, $\Delta F_{8O}^{\pm }=\Delta F_{9O}^{\pm }<0$ and $\Delta F_{2O}^{\pm }<0,$ which suggests that these atoms are sensitive to electrophilic reagents and are not conducive to electrophilic addition reactions but to capture H^+^. Therefore, the free-radical addition reaction should occur on C3 and C4.

As shown in Figure 4B, the free-radical reaction indices of C18 and C3 are $F_{18C}^{0}=$ 12.06972 and $F_{3C}^{0}=7.81404$. Since the value for C18 is higher than that for C3, the former is more prone to undergo free-radical polymerization than the latter. Meanwhile, $\Delta F_{18C}^{\pm }=$ −1.59224 and $\Delta F_{3C}^{\pm }=$ −8.01528; therefore, both C18 and C3 atoms are nucleophilic. The nucleophilicity of C3 is higher than that of C18, meaning that the nucleophilic reactions might occur on C3 and the free-radical reaction on C18.

As depicted in Figure 4C, $F_{12C}^{0}=\;$3.4096 and $F_{14C}^{0}=$ 10.3610, which clearly indicate that C14 is more susceptible to participate in free-radical polymerization. Moreover, the fact that $\Delta F_{12C}^{\pm }=$ 0.8156 and $\Delta F_{14C}^{\pm }=\;$1.3148 suggests that both atoms are electrophilic. As a result, both the free-radical reaction and the electrophilic addition should occur on C14.

As shown in Figure 4D, the free-radical reaction indices of C1, C2, and O18 are $F_{1C}^{0}=$ 5.1451, $F_{2C}^{0}=\;$2.3794, and $F_{18O}^{0}=\;$4.83023, indicating the highest susceptibility of C1 to undergo free-radical reactions. However, the difference between the indices of O18 and C1 is not significant; therefore, a free-radical reaction occurring on O18 cannot be ruled out. In addition, the values $\Delta F_{1C}^{\pm }=$ −7.88348, $\Delta F_{2C}^{\pm }=\;-$3.95462, and $\Delta F_{18O}^{\pm }=$ −7.29698 indicate that the three atoms are sensitive to electrophilic reagents. Owing to the stronger nucleophilicity of C1 and C18 possessing similar dual descriptor reactive indices, nucleophilic reactions are likely to occur on both atoms. It can be concluded that C1 and O18 have similar possibilities of participating in both free-radical reactions and nucleophilic reactions.

### Analysis of the Mechanism of Maleic Anhydride Ring-Opening Reaction

**Figure 5:**
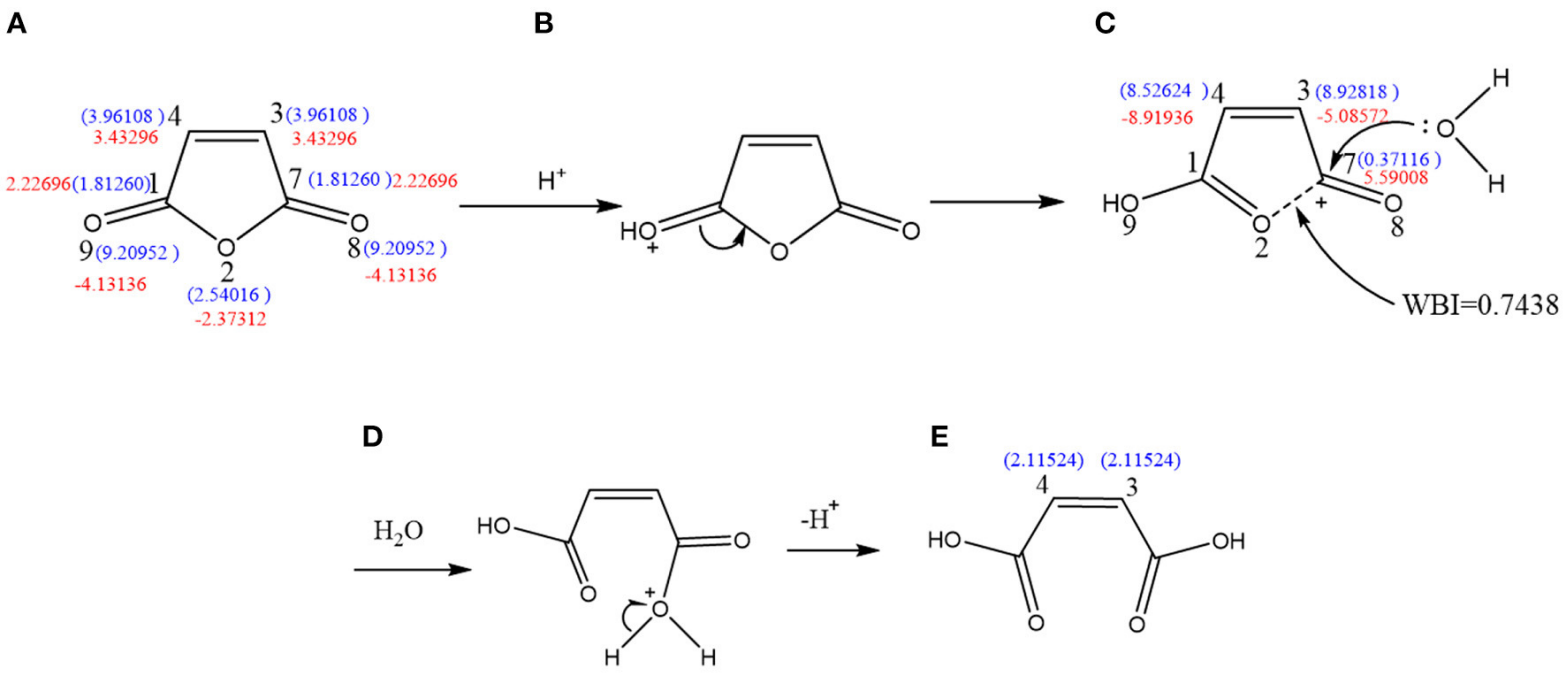
Molecule **(A)** possesses the values ${\text{F}}_{3c}^{0}$ = ${\text{F}}_{4C}^{0}$ = 3.96108 and ${\text{F}}_{8O}^{0}$ = ${\text{F}}_{9O}^{0}$ = 9.20952. Accordingly, in the presence of H^+^, oxygen atoms will trap hydrogen ions to form protonated **(B)**. Then, electron transfer occurs to form **(C)**. The free radical reaction indices of C3-c and C4-c were determined to be ${\text{F}}_{3C-c}^{0}$ = 8.92818 and ${\text{F}}_{4C-c}^{0}$ = 8.52624, indicating that the reactivity of free radicals is greatly enhanced. Therefore, the protonated MA radical reaction is the strongest, and C3-c or C4-c may participate in the radical polymerization reaction. The Wiberg bond index between O2-c-C7-c is WBIC-O = 0.7438, which is lower than the normal value of 0.954. This suggests that this bond is easy to break. The value δ ${\text{F}}_{7C-c}^{\pm }$ = 5.59008 indicates that C7-c has a strong electrophilicity. Then, the lone pair of electrons of the O atom in H_2_O attacks C7-c, causing the O2-c-C7-c bond to break **(D)**, resulting in the ring-opening of MA **(E)**.

As shown in Figure 5, molecule (a) possesses the values $F_{3C}^{0}=F_{4C}^{0}=\;$3.96108 and $F_{8O}^{0}=F_{9O}^{0}=\;$9.20952. Accordingly, in the presence of H^+^, oxygen atoms will trap hydrogen ions to form protonated MA (b). Then, electron transfer occurs to form structure (c). The free-radical reaction indices of C3-c and C4-c were determined to be $F_{3C-c}^{0}=\;$8.92818 and $F_{4C-c}^{0}=$ 8.52624, indicating that the reactivity of free radicals is greatly enhanced. Therefore, the protonated MA radical reaction is the strongest, and C3-c or C4-c may participate in the radical polymerization reaction. The Wiberg bond index between O2-c-C7-c is WBIC-O = 0.7438, which is lower than the normal value of 0.954. This suggests that this bond is easy to break. The value $\Delta F_{7C-c}^{\pm }=\;$5.59008 indicates that C7-c has a strong electrophilicity (c). Then, the lone pair of electrons of the O atom in H_2_O attacks C7-c, causing the O2-c-C7-c bond to break (d), resulting in the ring opening of MA.

### Effect of the Molar Ratios of Monomers on the Appearance of Epoxy–Acrylic Resin

**Figure 6:**
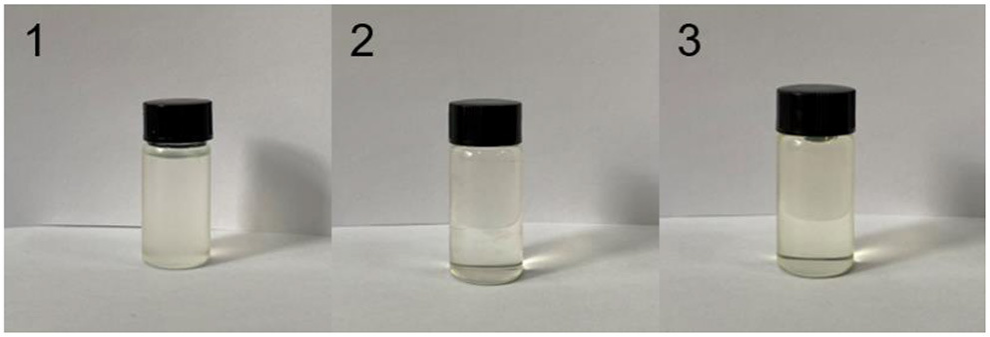
Influence of different monomer molar ratios on the appearance of epoxy–acrylic resin (EMPA).

Figure 6 shows that EMPA-1 has a turbid appearance with low light transmittance; that EMPA-2 is clear and transparent, slightly yellow, with strong light transmission; and that EMPA-3 is clear and yellow with low light transmittance. When the epoxy group concentration is high, the system is turbid; when the acid anhydride group concentration is high, the system is clear but exhibits darker colors. Accordingly, a ratio of epoxy group to acid anhydride of 0.5:0.4 provides a clear and colorless EMPA.

### Mechanics Performance Testing

As shown in Table 2, when the ratio of AGE:MA is 0.5:0.3, the tensile breaking strength, tensile strength, and bending strength of EMPA-1 are all high, but the hardness is one level lower. when the ratio of AGE:MA is 0.5:0.5, the hardness of EMPA-3 increases but the tensile breaking strength, tensile strength and bending strength decrease. when the ratio of AGE:MA is 0.5:0.4, the comprehensive mechanical properties of EMPA-2 are stronger than EMPA-1 and EMPA-3. Prove the scientificity of the calculated ratio.

### Scanning Electron Microscopy Images of Epoxy–Acrylic Resin

**Figure 7:**
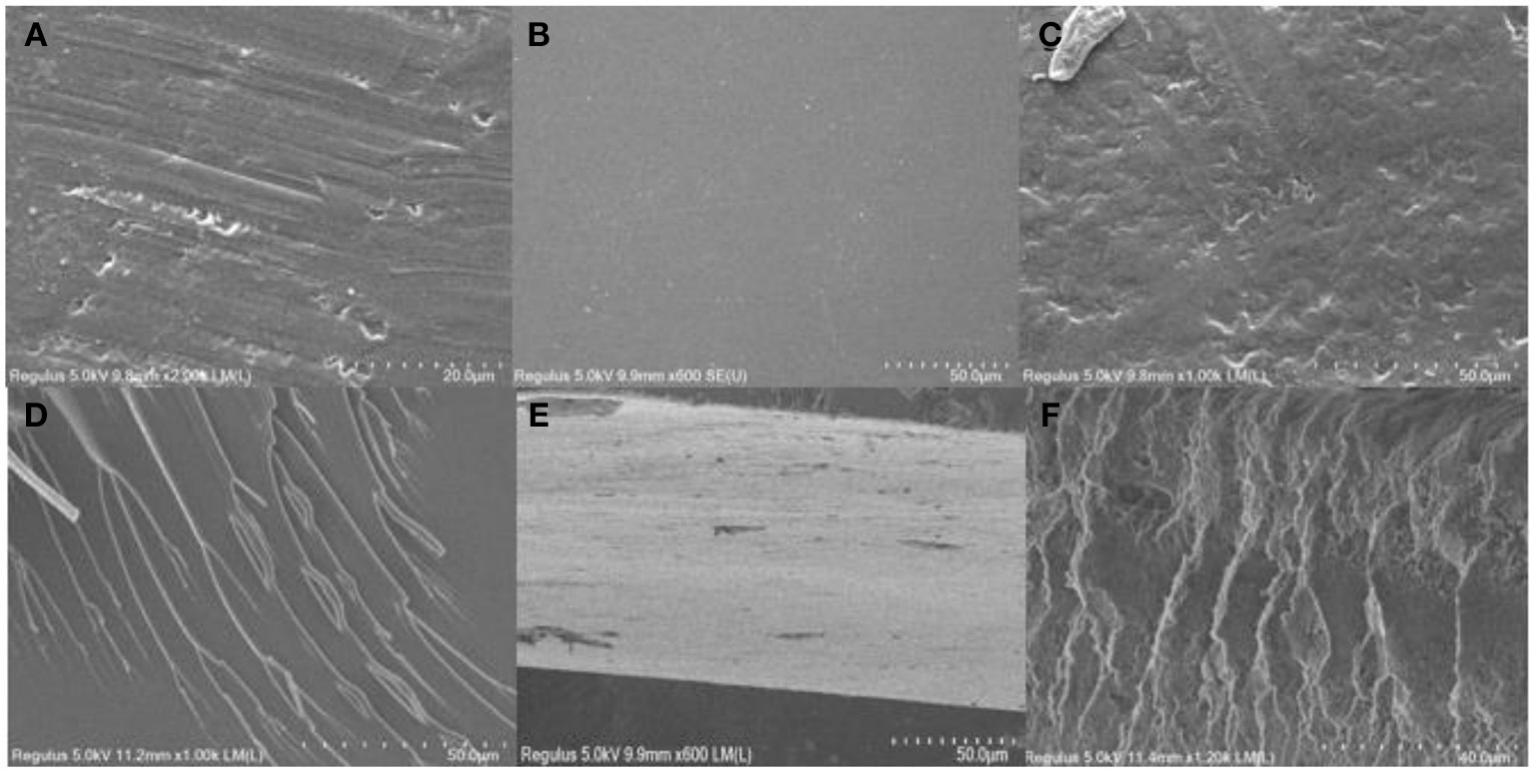
**(A)** Scanning electron microscopy (SEM) plan view of epoxy–acrylic resin (EMPA)-1. **(B)** SEM plan view of EMPA-2. **(C)** SEM plan view of EMPA-3. **(D)** SEM cross-sectional view of EMPA-1. **(E)** SEM cross-sectional view of EMPA-2. **(F)** SEM cross-sectional view of EMPA-3.

**Figure 8:**
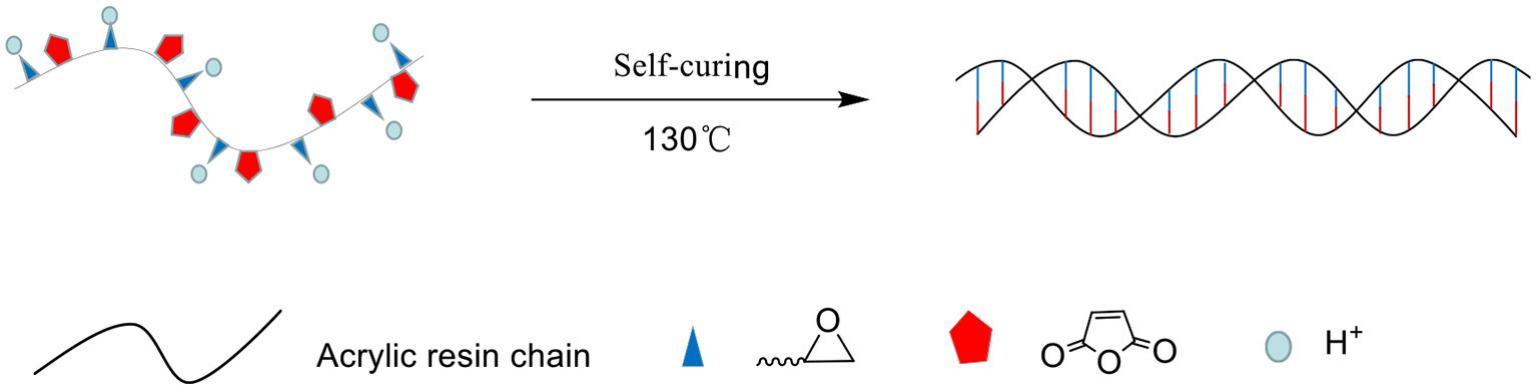
Epoxy–acrylic resin (EMPA) self-curing mechanism.

Figure 7 shows that self-cured EMPA is crosslinked in the superimposed manner depicted in Figure 8. Moreover, the coating surface roughness after curing follows the order EMPA-3 > EMPA-1 > EMPA-2, and the order of crosslink density is EMPA-2 > EMPA-1 > EMPA-3. As shown in Figures 7A,D, EMPA-1 has a rough surface, albeit with a somewhat regular texture. Since the number of epoxy groups in EMPA-1 is higher than that of acid anhydride groups, after EMPA-1 is cured, the acid anhydride group in the system is completely consumed, and the residual linear epoxy structure causes dents in the coating surface. As shown in Figures 7C,F, the epoxy group content is lower than that of acid anhydride groups in EMPA-3, resulting in the complete consumption of linear epoxy groups when EMPA-3 is cured. The remaining planar acid anhydride groups cause the coating surface to bulge, making the surface of EMPA-3 rough and irregular. EMPA-2 has a smooth surface and high crosslink density. From Figures 7B,D, it can be concluded that EMPA-2 has the most ideal proportion of epoxy and acid anhydride groups, and no reactive groups remain after curing.

### Thermogravimetry/Derivative Thermogravimetry Analysis of Epoxy–Acrylic Resin

**Figure 9:**
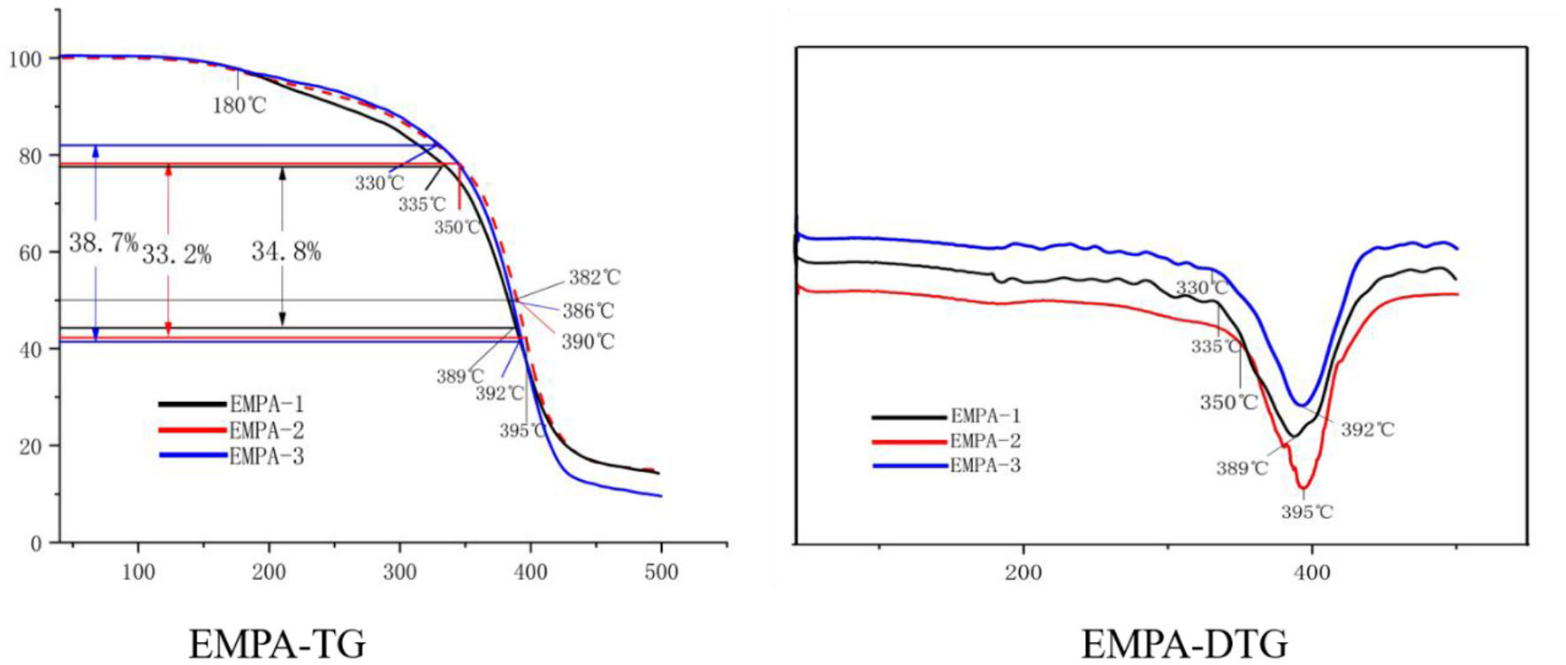
Thermogravimetry/derivative thermogravimetry (TG/DTG) of epoxy–acrylic resin (EMPA) with different monomer molar ratios.

As shown in Figure 9, during thermal decomposition, the first stage, which corresponds to a physical dehydration process, starts at room temperature and ends at 180°C. At 335°C, the main chain of EMPA-1 starts to split and lose weight, and a maximum weight loss rate of 6.42%/min is reached at 389°C. This process occurs at 350°C for EMPA-2, with a maximum weight loss rate of 7.38%/min at 395°C. For EMPA-3, the main chain starts to split and lose weight at 330°C, and a maximum weight loss rate of 6.24%/min is reached at 392°C. From these results, it can be inferred that EMPA-1 remains stable below 335°C, EMPA-2 below 350°C, and EMPA-3 below 330°C. As can be extracted from the EMPA-TG diagram, the half-life temperature of EMPA-2 is higher than that of the other two. According to the initial decomposition temperature, the thermal stability of EMPA-2 is higher than that of EMPA-1 and EMPA-3.

## Conclusions

According to the CDFT and the formula involved in *Density Functional Theory Calculation of the Radical Reaction Index of Each Reactive Monomer*, the quantum chemical reactivity index of each molecule is calculated. Obtain the free-radical reactivity index and double descriptor reactivity index of each molecule involved in this article. According to the radical reactivity index of each atom in the molecule, the atoms participating in the reaction during radical polymerization are found. The nucleophilic index or electrophilic index of the atom at the site is quantitatively obtained by the dual descriptor reactivity index to determine the rationality of the reaction. Judge the structure of MA after the reaction based on the size of the Wiberg bond index. Therefore, it is scientific to infer the molar ratio of each reactant by obtaining the quantum chemical reactivity index of the reactant molecules in the radical polymerization reaction. According to the reactivity index of the functional group atoms in each reactant, the molar ratio of each reactant in free-radical polymerization was deduced, and a self-curing thermosetting EMPA was synthesized. EMPA is stable at room temperature and can be self-cured by heating according to the following mechanism: at high temperature, H^+^ attacks the epoxy group, which opens to form a new reactive group. The obtained reactive groups undergo crosslinking reactions with the acid anhydride groups present in the system. The EMPA resin synthesized at 80°C can be diluted by butyl acetate to solid content of 50%, and the solution can be stored for over 1 year without gellation (Chinese standards/GB 6753.3-86). In addition, EMPA can be added to epoxy resin as a latent curing agent to form an epoxy resin self-curing system.

## Data Availability Statement

The original contributions presented in the study are included in the article/Supplementary Material, further inquiries can be directed to the corresponding author.

## Author Contributions

QA came up with ideas and design experiments. BY implemented specific experiments. All authors contributed to the article and approved the submitted version.

## Conflict of Interest

The authors declare that the research was conducted in the absence of any commercial or financial relationships that could be construed as a potential conflict of interest.
