# Supplementary material for: Density Functional Theory-Assisted Synthesis of Self-Curing Epoxy–Acrylic Resin
Source: Front Chem. 2021 Jan 20;8:595954. doi: 10.3389/fchem.2020.595954 (PMC7855980; doi:10.3389/fchem.2020.595954)
Supplement: Supplementary file 1 [file Data_Sheet_1.docx]

**maleic anhydride**


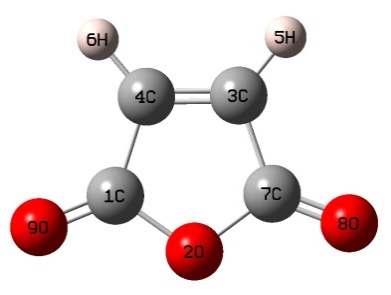


**Global Reactivity Index**

| **Basis set** | B3LYP/6311g++(2d, 2p）  /gas | | Single point energy  E  （au） | Valence electron number  *Ve* |  |
| --- | --- | --- | --- | --- | --- |
|  |  |  |  |  |  |
| **Energy**  **form** | $E^{0}$ | N | -379.416 | 36 |  |
|  | $E^{+1e}$ | N+1 | -379.439 |  |  |
|  | $E^{-1e}$ | N-1 | -378.985 |  |  |
| **Global Index** | I |  | 0.430858 |  |  |
|  | A |  | 0.023901 |  |  |
|  | μ |  | -0.22738 |  |  |
|  | η |  | 0.203478 |  |  |
|  | ω |  | 0.127044 |  |  |

**Local reactivity index**

| Ato. No. | $q_{k}^{N}$ | $q_{k}^{N+1}$ | $q_{k}^{N-1}$ | $f_{k}^{+}$ | $f_{k}^{-}$ | $f_{k}^{0}$ | $Ve\Delta\omega_{k}^{\pm}$ | $Vef_{k}^{0}$ |
| --- | --- | --- | --- | --- | --- | --- | --- | --- |
| C1 | 0.7770 | 0.6958 | 0.7965 | 0.0813 | 0.0194 | 0.0504 | 0.2829 | 1.8126 |
| O2 | -0.5567 | -0.5943 | -0.4532 | 0.0376 | 0.1035 | 0.0706 | -0.3015 | 2.5402 |
| C3 | -0.2294 | -0.3871 | -0.1671 | 0.1577 | 0.0624 | 0.1100 | 0.4361 | 3.9611 |
| C4 | -0.2294 | -0.3871 | -0.1671 | 0.1577 | 0.0624 | 0.1100 | 0.4361 | 3.9611 |
| H5 | 0.2333 | 0.1895 | 0.2865 | 0.0438 | 0.0533 | 0.0485 | -0.0434 | 1.7464 |
| H6 | 0.2333 | 0.1895 | 0.2865 | 0.0438 | 0.0533 | 0.0485 | -0.0434 | 1.7464 |
| C7 | 0.7770 | 0.6958 | 0.7965 | 0.0813 | 0.0194 | 0.0504 | 0.2829 | 1.8126 |
| O8 | -0.5025 | -0.7010 | -0.1893 | 0.1984 | 0.3132 | 0.2558 | -0.5249 | 9.2095 |
| O9 | -0.5025 | -0.7010 | -0.1893 | 0.1984 | 0.3132 | 0.2558 | -0.5249 | 9.2095 |

**Butyl Acrylate**


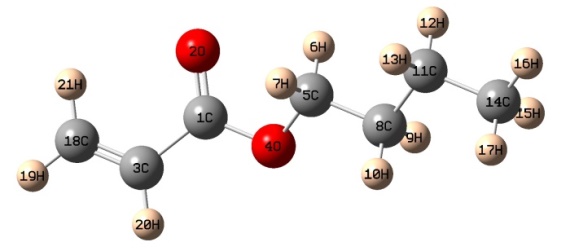


**Global Reactivity Index**

| **Basis set** | B3LYP/6311g++(2d, 2p）  /gas | | Single point energy  E  （au） | Valence electron number  *Ve* |  |
| --- | --- | --- | --- | --- | --- |
|  |  |  |  |  |  |
| **Energy**  **form** | $E^{0}$ | N | -424.5573 | 52 |  |
|  | $E^{+1e}$ | N+1 | -424.5506 |  |  |
|  | $E^{-1e}$ | N-1 | -424.1833 |  |  |
| **Global Index** | I |  | 0.3740 |  |  |
|  | A |  | -0.0067 |  |  |
|  | μ |  | -0.1837 |  |  |
|  | η |  | 0.1904 |  |  |
|  | ω |  | 0.0886 |  |  |

**Local reactivity index**

| Ato. No. | $q_{k}^{N}$ | $q_{k}^{N+1}$ | $q_{k}^{N-1}$ | $f_{k}^{+}$ | $f_{k}^{-}$ | $f_{k}^{0}$ | $\Delta\omega_{k}^{\pm}$ | $Ve\Delta\omega_{k}^{\pm}$ | $Vef_{k}^{0}$ |
| --- | --- | --- | --- | --- | --- | --- | --- | --- | --- |
| C1 | 0.7832 | 0.7194 | 0.7376 | 0.0638 | -0.0455 | 0.0091 | 0.0097 | 0.5035 | 0.4740 |
| O2 | -0.6125 | -0.6979 | -0.5245 | 0.0854 | 0.0880 | 0.0867 | -0.0002 | -0.0118 | 4.5094 |
| C3 | -0.2980 | -0.3712 | -0.0707 | 0.0732 | 0.2273 | 0.1503 | -0.0137 | -0.7100 | 7.8140 |
| O4 | -0.5585 | -0.5839 | -0.4913 | 0.0253 | 0.0672 | 0.0463 | -0.0037 | -0.1929 | 2.4066 |
| C5 | -0.0208 | -0.0191 | -0.0330 | -0.0017 | -0.0121 | -0.0069 | 0.0009 | 0.0482 | -0.3593 |
| H6 | 0.1822 | 0.1695 | 0.2329 | 0.0127 | 0.0507 | 0.0317 | -0.0034 | -0.1752 | 1.6497 |
| H7 | 0.1822 | 0.1715 | 0.2329 | 0.0107 | 0.0507 | 0.0307 | -0.0035 | -0.1843 | 1.5959 |
| C8 | -0.3945 | -0.3994 | -0.4020 | 0.0049 | -0.0075 | -0.0013 | 0.0011 | 0.0568 | -0.0684 |
| H9 | 0.1949 | 0.1838 | 0.2440 | 0.0111 | 0.0491 | 0.0301 | -0.0034 | -0.1751 | 1.5639 |
| H10 | 0.1949 | 0.1878 | 0.2440 | 0.0071 | 0.0491 | 0.0281 | -0.0037 | -0.1935 | 1.4602 |
| C11 | -0.3768 | -0.3882 | -0.3779 | 0.0113 | -0.0010 | 0.0051 | 0.0011 | 0.0568 | 0.2678 |
| H12 | 0.1866 | 0.1616 | 0.2305 | 0.0250 | 0.0439 | 0.0345 | -0.0017 | -0.0872 | 1.7914 |
| H13 | 0.1866 | 0.1673 | 0.2306 | 0.0194 | 0.0439 | 0.0316 | -0.0022 | -0.1131 | 1.6455 |
| C14 | -0.5640 | -0.5948 | -0.5652 | 0.0308 | -0.0012 | 0.0148 | 0.0028 | 0.1473 | 0.7706 |
| H15 | 0.1927 | 0.1077 | 0.2175 | 0.0850 | 0.0249 | 0.0549 | 0.0053 | 0.2770 | 2.8561 |
| H16 | 0.1997 | 0.0143 | 0.2227 | 0.1854 | 0.0229 | 0.1042 | 0.0144 | 0.7486 | 5.4176 |
| H17 | 0.1927 | 0.1265 | 0.2175 | 0.0662 | 0.0249 | 0.0455 | 0.0037 | 0.1903 | 2.3665 |
| C18 | -0.2753 | -0.4921 | -0.0279 | 0.2168 | 0.2474 | 0.2321 | -0.0027 | -0.1410 | 12.0697 |
| H19 | 0.1905 | 0.1654 | 0.2186 | 0.0252 | 0.0281 | 0.0266 | -0.0003 | -0.0132 | 1.3845 |
| H20 | 0.2093 | 0.1832 | 0.2358 | 0.0261 | 0.0265 | 0.0263 | 0.0000 | -0.0019 | 1.3676 |
| H21 | 0.2051 | 0.1887 | 0.2278 | 0.0164 | 0.0227 | 0.0196 | -0.0006 | -0.0293 | 1.0171 |

**Styrene**


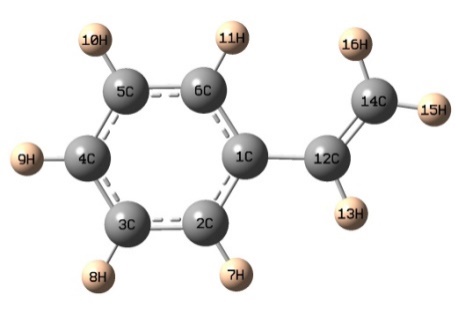


**Global Reactivity Index**

| **Basis set** | B3LYP/6311g++(2d, 2p）  /gas | | Single point energy  E  （au） | Valence electron number  *Ve* |  |
| --- | --- | --- | --- | --- | --- |
|  |  |  |  |  |  |
| **Energy**  **form** | $E^{0}$ | N | -309.7435 | 40 |  |
|  | $E^{+1e}$ | N+1 | -309.7294 |  |  |
|  | $E^{-1e}$ | N-1 | -309.4386 |  |  |
| **Global index** | I |  | 0.3049 |  |  |
|  | A |  | -0.0141 |  |  |
|  | μ |  | -0.1454 |  |  |
|  | η |  | 0.1595 |  |  |
|  | ω |  | 0.0663 |  |  |

**Local reactivity index**

| Ato. No. | $q_{k}^{N}$ | $q_{k}^{N+1}$ | $q_{k}^{N-1}$ | $f_{k}^{+}$ | $f_{k}^{-}$ | $f_{k}^{0}$ | $\Delta\omega_{k}^{\pm}$ | $Ve\Delta\omega_{k}^{\pm}$ | $Vef_{k}^{0}$ |
| --- | --- | --- | --- | --- | --- | --- | --- | --- | --- |
| C1 | -0.0811 | -0.1523 | 0.0217 | 0.0712 | 0.1028 | 0.0870 | -0.0021 | -0.0837 | 3.4798 |
| C2 | -0.1804 | -0.2099 | -0.1142 | 0.0295 | 0.0662 | 0.0479 | -0.0024 | -0.0974 | 1.9142 |
| C3 | -0.1974 | -0.2611 | -0.1688 | 0.0637 | 0.0286 | 0.0461 | 0.0023 | 0.0932 | 1.8452 |
| C4 | -0.1995 | -0.3612 | -0.0174 | 0.1616 | 0.1821 | 0.1719 | -0.0014 | -0.0543 | 6.8744 |
| C5 | -0.1942 | -0.2007 | -0.1778 | 0.0065 | 0.0164 | 0.0115 | -0.0007 | -0.0261 | 0.4582 |
| C6 | -0.1789 | -0.2884 | -0.1130 | 0.1095 | 0.0658 | 0.0877 | 0.0029 | 0.1159 | 3.5072 |
| H7 | 0.1999 | 0.1734 | 0.2317 | 0.0264 | 0.0318 | 0.0291 | -0.0004 | -0.0143 | 1.1648 |
| H8 | 0.2032 | 0.1708 | 0.2408 | 0.0325 | 0.0375 | 0.0350 | -0.0003 | -0.0134 | 1.4006 |
| H9 | 0.2030 | 0.1709 | 0.2344 | 0.0321 | 0.0314 | 0.0318 | 0.0000 | 0.0017 | 1.2706 |
| H10 | 0.2024 | 0.1714 | 0.2398 | 0.0310 | 0.0374 | 0.0342 | -0.0004 | -0.0169 | 1.3672 |
| H11 | 0.2000 | 0.1844 | 0.2250 | 0.0156 | 0.0250 | 0.0203 | -0.0006 | -0.0250 | 0.8116 |
| C12 | -0.1894 | -0.2605 | -0.1387 | 0.0711 | 0.0507 | 0.0609 | 0.0014 | 0.0541 | 2.4354 |
| H13 | 0.1909 | 0.1581 | 0.2247 | 0.0329 | 0.0338 | 0.0333 | -0.0001 | -0.0024 | 1.3336 |
| C14 | -0.3503 | -0.6258 | -0.1077 | 0.2755 | 0.2426 | 0.2590 | 0.0022 | 0.0872 | 10.3610 |
| H15 | 0.1922 | 0.1646 | 0.2218 | 0.0276 | 0.0295 | 0.0286 | -0.0001 | -0.0051 | 1.1432 |
| H16 | 0.1796 | 0.1663 | 0.1980 | 0.0133 | 0.0184 | 0.0158 | -0.0003 | -0.0135 | 0.6332 |

**Propenyl glycidyl ether**


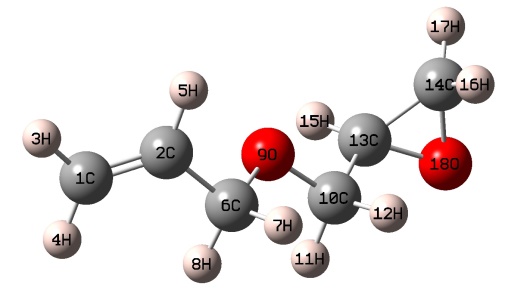


**Global Reactivity Index**

| **Basis set** | B3LYP/6311g++(2d, 2p）  /gas | | Single point energy  E  （au） | Valence electron number  *Ve* |  |
| --- | --- | --- | --- | --- | --- |
|  |  |  |  |  |  |
| **Energy**  **form** | $E^{0}$ | N | -385.1454 | 46 |  |
|  | $E^{+1e}$ | N+1 | -385.1251 |  |  |
|  | $E^{-1e}$ | N-1 | -384.8022 |  |  |
| **Global index** | I |  | 0.3432 |  |  |
|  | A |  | -0.0203 |  |  |
|  | μ |  | -0.1614 |  |  |
|  | η |  | 0.1818 |  |  |
|  | ω |  | 0.0717 |  |  |

**Local reactivity index**

| Ato. No. | $q_{k}^{N}$ | $q_{k}^{N+1}$ | $q_{k}^{N-1}$ | $f_{k}^{+}$ | $f_{k}^{-}$ | $f_{k}^{0}$ | $\Delta\omega_{k}^{\pm}$ | $Ve\Delta\omega_{k}^{\pm}$ | $Vef_{k}^{0}$ |
| --- | --- | --- | --- | --- | --- | --- | --- | --- | --- |
| C1 | -0.3600 | -0.3861 | -0.1624 | 0.0262 | 0.1975 | 0.1119 | -0.0123 | -0.5652 | 5.1451 |
| C2 | -0.1839 | -0.1926 | -0.0892 | 0.0087 | 0.0947 | 0.0517 | -0.0062 | -0.2835 | 2.3794 |
| H3 | 0.1919 | 0.0696 | 0.2192 | 0.1222 | 0.0273 | 0.0748 | 0.0068 | 0.3131 | 3.4397 |
| H4 | 0.1818 | 0.0649 | 0.2011 | 0.1169 | 0.0193 | 0.0681 | 0.0070 | 0.3217 | 3.1331 |
| H5 | 0.1924 | 0.1179 | 0.2190 | 0.0744 | 0.0266 | 0.0505 | 0.0034 | 0.1577 | 2.3244 |
| C6 | -0.0461 | -0.0741 | -0.0908 | 0.0280 | -0.0446 | -0.0083 | 0.0052 | 0.2395 | -0.3832 |
| H7 | 0.1672 | 0.0611 | 0.2506 | 0.1062 | 0.0834 | 0.0948 | 0.0016 | 0.0750 | 4.3597 |
| H8 | 0.1627 | 0.0745 | 0.2182 | 0.0882 | 0.0555 | 0.0719 | 0.0023 | 0.1079 | 3.3051 |
| O9 | -0.6035 | -0.6142 | -0.4401 | 0.0106 | 0.1635 | 0.0871 | -0.0110 | -0.5041 | 4.0043 |
| C10 | -0.0335 | -0.0429 | -0.0583 | 0.0094 | -0.0247 | -0.0077 | 0.0024 | 0.1125 | -0.3537 |
| H11 | 0.1653 | 0.1275 | 0.2140 | 0.0378 | 0.0487 | 0.0432 | -0.0008 | -0.0358 | 1.9893 |
| H12 | 0.1635 | 0.1115 | 0.2166 | 0.0520 | 0.0531 | 0.0526 | -0.0001 | -0.0037 | 2.4180 |
| C13 | 0.0815 | 0.0789 | 0.0645 | 0.0026 | -0.0171 | -0.0072 | 0.0014 | 0.0650 | -0.3321 |
| C14 | -0.0565 | -0.0767 | -0.0483 | 0.0203 | 0.0082 | 0.0142 | 0.0009 | 0.0398 | 0.6537 |
| H15 | 0.1822 | 0.1533 | 0.2288 | 0.0289 | 0.0466 | 0.0377 | -0.0013 | -0.0585 | 1.7358 |
| H16 | 0.1745 | 0.0596 | 0.2045 | 0.1148 | 0.0300 | 0.0724 | 0.0061 | 0.2798 | 3.3316 |
| H17 | 0.1781 | 0.0510 | 0.2258 | 0.1271 | 0.0477 | 0.0874 | 0.0057 | 0.2617 | 4.0202 |
| O18 | -0.5575 | -0.5832 | -0.3732 | 0.0257 | 0.1843 | 0.1050 | -0.0114 | -0.5231 | 4.8302 |
